# Laboratory-Developed Tests: Design of a Regulatory Strategy in Compliance with the International State-of-the-Art and the Regulation (EU) 2017/746 (EU IVDR [In Vitro Diagnostic Medical Device Regulation])

**DOI:** 10.1007/s43441-021-00323-7

**Published:** 2021-07-21

**Authors:** Folker Spitzenberger, Jaimin Patel, Inga Gebuhr, Klaus Kruttwig, Abdulrahim Safi, Christian Meisel

**Affiliations:** 1 Centre for Regulatory Affairs in Biomedical Sciences, Technische Hochschule Lübeck, Mönkhofer Weg 239, 23562 Lübeck, Germany; 2 Labor Berlin - Charité Vivantes GmbH, Sylter Strasse 2, 13353 Berlin, Germany; 3 Leibniz Institute of Photonic Technology E.V. Jena, Albert-Einstein-Str. 9, 07745 Jena, Germany

**Keywords:** Laboratory-developed tests, LDT, In-house IVD, IVDR, Validation, Conformity assessment

## Abstract

**Purpose:**

This study aimed at the development of a regulatory strategy for compliance of laboratory-developed tests (LDTs) with requirements of the Regulation (EU) 2017/746 (“EU-IVDR”) under consideration of international requirements for LDTs as established in major regulatory regions. Furthermore, it was analysed in how far elements of current LDT regulation could qualify for an internationally harmonised concept ensuring quality, safety and performance of LDTs.

**Methods:**

A review of regulatory literature including legislation as well as guidance documents was performed. The regulatory strategy was adapted from international guidance concepts used for commercially marketed IVD. It was then applied to the example of a large medical laboratory in the EU. A high-level comparison was conducted to identify gaps and matches between the different international regulatory requirements for LDTs.

**Results:**

A four-step strategy for compliance of LDTs with the EU IVDR was implemented in an exemplary medical laboratory. On the basis of an internationally used LDT definition, LDTs constitute nearly 50% of the total IVD devices used in the laboratory. While an ISO 15189-compliant QMS is a major component, it should be accompanied by the application of appropriate processes for risk management, performance evaluation and continuous monitoring of LDTs. At least six criteria represent common characteristics of a potential, internationally convergent concept for the regulation/standardization of LDTs.

**Conclusions:**

This study confirms the impact of LDTs for individualized and innovative medical laboratory testing. Prerequisites for LDT use as especially given by the IVDR and missing interpretation in the EU with regard to the scope of LDT definition, the application of standards and the extent of documentation for LDTs currently lead to uncertainties for both laboratories and regulatory bodies responsible for LDT oversight. The characteristics identified as common criteria for ensuring quality, safety and performance of LDTs may be considered as central elements of future international consensus guidance.

## Introduction

Laboratory developed tests (LDTs) or “in-house IVD” are broadly used in medical laboratories. LDTs are in vitro diagnostic testing methods that are performed by using in vitro diagnostic medical devices (IVD) which are developed, manufactured, and used within a single health institution and its corresponding laboratory. Normally, they are not available on an industrial scale.

The medical relevance of LDTs is highly significant though, because LDTs are used for patient diagnosis and monitoring in a wide scope of rare and/or emerging diseases in almost all fields of medical laboratory testing [1]. Most recently, the significance of LDTs has been proven by their substantial input for the detection of SARS-CoV-2 infection markers and, therefore, during the management of the COVID-19 pandemic [2]. From the quantitative point of view, LDTs also hold a significant market share in the global field of medical laboratory testing. Consequently, the setting of quality, safety and performance requirements within a regulatory framework for LDTs is relevant and is expected to have a significant impact on both individual patient care and on public health and safety [3, 4].

However, there is a controversial regulatory debate with regard to LDTs, especially in regions with established regulatory systems for medical devices and IVD. While Australia has implemented an advanced regulatory system for LDTs within the last decade, the United States are currently in the process of reforming their current regulatory framework related to LDTs [5–8]. In Canada, LDTs are currently not regulated on the national level and the individual provinces and territories are responsible for the delivery and administration of health care services including LDTs [9, 10].

In the European Union (EU), LDTs are exempted from all of the requirements under the current Directive 98/79/EC (in vitro diagnostic Directive—IVDD) [11]. EU Member states have the right to create national provisions subjecting LDTs under appropriate protective measures. While some countries, for example Germany and Austria, currently regulate LDTs within the scope of their national medical device acts [12, 13], other EU members may not provide any regulatory framework, leading to the criticism that the exemption under the IVDD does not ensure a uniform safety and performance level for LDTs across Europe [14]. With the new Regulation (EU) 2017/746 (“EU IVDR”) that was published in May 2017 and will fully apply from 26 May 2022, the EU will for the first time set harmonized requirements for IVD that are manufactured and used in the same health institution. These requirements are set out according to Article 5 paragraph 5 of the IVDR [15]. As this regulation does not need to be transposed into national law, one may assume that it will not only introduce an EU-wide regulatory framework but will also reduce the risks of discrepancies in interpretation across the EU.

However, many uncertainties and even urgent questions remain, not only for the laboratories affected by the new EU requirements, but also for the regulatory authorities that are supposed to monitor the compliance with the EU IVDR in the future. These questions address such fundamental aspects as, for example, the definition of the scope of the IVDR requirements for self-developed testing systems, the extent of the required quality and risk management system, the acceptable elements and limits for a justification of LDT use in comparison with the use of commercially available IVD, the requirements for performance evaluation and its subsequent documentation [16–18].

Despite considerable international efforts for harmonising regulatory requirements for medical devices, as driven, for example, by the International Medical Device Regulators Forum (IMDRF] [19, 20], there is currently no common understanding or internationally harmonised approach for the compliance with minimum or essential requirements for LDTs available.

It was therefore the aim of this study to develop a regulatory and quality strategy for compliance of LDTs with requirements according to the EU IVDR under consideration of certain international requirements for LDTs as established in major regulatory markets.

On the basis of this strategy, it was further analysed in how far essential regulatory elements for LDTs could be identified on a global basis to represent key characteristics of a regulatory, internationally convergent concept for ensuring quality, safety and performance of LDTs.

## Materials and Methods

To obtain a clear understanding of the background of the subject, a detailed literature review was performed. The collection, selection and analysis of the relevant regulatory literature followed a priority order as suggested by WHO for the hierarchy of regulation [20].

Therefore, the literature was differentiated into current and prospectively applicable legislation (primary and secondary legislation which is mandatory to be fulfilled) and guidelines that generally refer to non-binding documents issued by regulatory authorities, which offer guidance on recommended practices. A special focus was also laid on standards, since the application of recognized or (in the EU) “harmonized” standards is understood in many regulatory markets as the most preferred way to gain compliance with regulatory requirements [21]. Standards are mostly not issued by regulatory authorities, but by standardization organizations [22, 23]. In addition to national, European and international standards, further international guidance documents were also identified and analyzed that can help to determine the regulatory requirements accepted at the international level and to recognize the state-of-the-art requirements.

Although the scope of the study was focused on the EU market in order to develop a concept to comply with the IVDR requirements for LDT, the analysis included the current regulatory situation for LDTs in further major and well-established medical device/IVD markets. To allow for comparison, the regulatory framework for LDTs in Australia, in the United States of America (U.S.) and in Canada was considered apart from the situation in the EU.

The methodology for the development of the EU IVDR compliance strategy for LDTs was adapted and modified from the major steps that international guidance documents suggest as regulatory strategy to lawfully place a device on a commercial market [20, 24, 25].

The strategy developed in this study was applied to the example of the large, academic medical laboratory Labor Berlin—Charité Vivantes GmbH (Labor Berlin), Berlin, Germany. The laboratory unites nine different departments under a single management and offers a comprehensive range of medical laboratory services with more than 70 million laboratory examinations per annum. The laboratory portfolio includes approximately 1300 examination procedures accredited according to EN ISO 15189 [26].

A high-level comparison was conducted with the aim to identify areas of compliance and of non-compliance (gaps) between the different regulatory markets and related requirements for LDTs.

## Results

### Development and Implementation of the EU IVDR Compliance Strategy for LDTs

**Figure 1:**
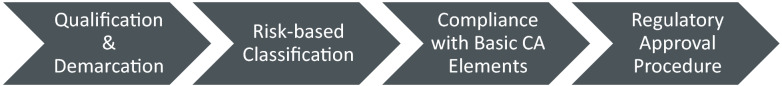
The four major steps of a regulatory strategy for medical devices: (1) qualification and demarcation of the considered product on the basis of applicable definitions, (2) risk-based classification of the device, (3) identification and fulfilment of basic elements of conformity assessment (CA), and (4) choice and conduct of the applicable regulatory approval procedure.

Internationally recognized elements related to conformity assessment of medical devices including IVD usually consist of at least four major steps as shown in Fig. 1 [20, 27]. For the purpose of this study, these elements were adapted to the special regulatory situation for LDTs as presented below.

### Applicable Definitions, Qualification, and Demarcation of LDTs

Whether an IVD is subject to IVD regulations or not, substantially depends on the intended purpose of the device that is defined by the manufacturer. The manufacturer is therefore responsible for the demarcation and subsequent activities leading to regulatory compliance of a device. In case of LDTs, the health institutions and/or related laboratories are the manufacturers and they are responsible for the aforementioned tasks.

While the EU IVDR according to Article 2 largely follows the international consensus with regard to the general definition of IVD [25, 28] (compare Table 1), it is of note that it does not include an autonomous definition of LDTs at all.

**Table 1:** The Definition of IVD According to the EU IVDR is Largely Convergent with the International Consensus as Originally Defined by GHTF and Adopted by IMDRF.

Regulation (EU) 2017/746 (IVDR)	GHTF/SG1/N071:2012 (IMDRF/IVD WG/N64FINAL:2021)
‘In Vitro diagnostic medical device’ means any medical device which is a reagent, reagent product, calibrator, control material, kit, instrument, apparatus, piece of equipment, software or system, whether used alone or in combination, intended by the manufacturer to be used in vitro for the examination of specimens, including blood and tissue donations, derived from the human body, solely or principally for the purpose of providing information on one or more of the following: (a) concerning a physiological or pathological process or state; (b) concerning congenital physical or mental impairments; (c) concerning the predisposition to a medical condition or a disease; (d) to determine the safety and compatibility with potential recipients; (e) to predict treatment response or reactions; (f) to define or monitoring therapeutic measures. Specimen receptacles shall also be deemed to be in vitro diagnostic medical devices;	‘In Vitro Diagnostic (IVD) medical device’ means a medical device, whether used alone or in combination, intended by the manufacturer for the in-vitro examination of specimens derived from the human body solely or principally to provide information for diagnostic, monitoring or compatibility purposes Note 1: IVD medical devices include reagents, calibrators, control materials, specimen receptacles, software, and related instruments or apparatus or other articles and are used, for example, for the following test purposes: diagnosis, aid to diagnosis, screening, monitoring, predisposition, prognosis, prediction, determination of physiological status Note 2: In some jurisdictions, certain IVD medical devices may be covered by other regulations

In fact, the EU IVDR does not even use the term „laboratory-developed test “or” “LDT”. According to Article 5 (5), the IVDR mentions “devices manufactured and used only within health institutions established in the Union”. Recital No. 29 of the IVDR sheds some light on the meaning of the term when considering that “health institutions should have the possibility of manufacturing, modifying and using devices in-house and thereby addressing, on a non-industrial scale, the specific needs of target patient groups which cannot be met at the appropriate level of performance by an equivalent device available on the market. …”.

While further official explanations related to LDTs in the context of the EU IVDR (as, for example, provided by the Medical Devices Coordination Group (MDCG)) are currently not existent, some interpretation is provided by the anterior European MEDDEV documents MEDDEV 2.14/1 and 2.14/2 relating to IVDD interpretation [29, 30]. MEDDEV 2.14/2 demarcates so-called “Research Use Only” (RUO) products from devices for performance evaluation, from devices manufactured and used only within the same health institution and from further uses in the context of research or IVD design and development. In summary, MEDDEV 2.14/2 concludes that for a product to be categorized as a RUO product it must have no intended medical purpose or objective.

Conversely, one may conclude that all devices used by a medical laboratory for an in vitro diagnostic medical purpose—even though they are not originally labelled as such by the original supplier and regardless of whether they are used alone or in combination– should be regarded as LDTs.

MEDDEV 2.14/1 supports this view by explaining that the qualification of a product as IVD, on the basis of its characteristics, is solely dependent on the manufacturer’s intention that the product should be used for in vitro diagnostic examination.

At the international level, the U. S. Food and Drug Administration (U. S. FDA) describes LDTs, previously known as “home brew tests”, as follows: “A laboratory developed test (LDT) is a type of in vitro diagnostic test that is intended for clinical use and designed, manufactured and used within a single laboratory” [1].

The Therapeutic Goods Administration (TGA) Australia recognizes LDTs as ‘In-house IVDs’ and defines as follows: “In-house IVDs are pathology tests that have been developed (or modified) within a laboratory (or laboratory network) to carry out testing on human samples, where the results are intended to assist in clinical diagnosis or be used in making decisions concerning clinical management” [31]. According to the Australian Therapeutic Goods (Medical Devices) Regulations 2002, a laboratory generally develops LDTs in three different ways: (1) LDTs developed from first principles; (2) LDTs developed or modified from a published source including those marked RUO, “investigational use only” (IUO) or “analyte specific reagent” (ASR); 3) LDTs developed by modifications to commercially supplied IVDs [31, 32].

In Canada, LDTs are currently not regulated by Health Canada and therefore, a national, legally binding definition is not existent [10]. However, the Canadian standard Z316.8-1 defines LDTs as “a test developed (or modified) and used within a single laboratory to carry out testing on samples, where the results are intended to assist in clinical diagnosis or be used in making decisions concerning clinical management.” In addition to the Australian explanations, the Canadian standard points out that laboratories are also considered to have developed an LDT, if “LDTs [are] used for a purpose other than the intended purpose assigned by the manufacturer—In vitro diagnostic devices become a laboratory-developed test when the intended use is different than the intended use claimed by the manufacturer; a physical component of the commercial IVDD is modified, substituted, or removed; or the test is not used in accordance with the manufacturer’s instructions for use” [33].

**Figure 2:**
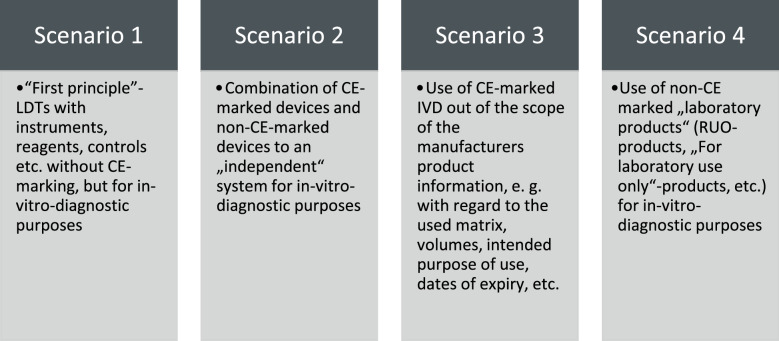
Although the IVDR does not include a definition of LDTs, there are four major scenarios for the qualification and demarcation of “devices manufactured and used only within health institutions” based on the laboratory’s intention that the product or product combination is intended to be used for in vitro diagnostic examination purposes.

Considering the regulatory demarcation for LDTs under this perspective, our study suggests that “devices manufactured and used only within health institutions” should be qualified as LDTs according to four major scenarios as illustrated in Fig. 2. The central qualification and demarcation element is the laboratory’s intention that the product should be used for in vitro diagnostic examination.

Since the intended purpose of a device is the basis for the qualification and demarcation process, it needs to be unambiguous, consistent and with details that can guide in the process of demarcation and also during subsequent risk classification. A corresponding tool was therefore developed according to Table 2 for the determination of the intended purpose of LDTs according to Annex I, Sect. 20.4.1 of the EU IVDR.

**Table 2:**
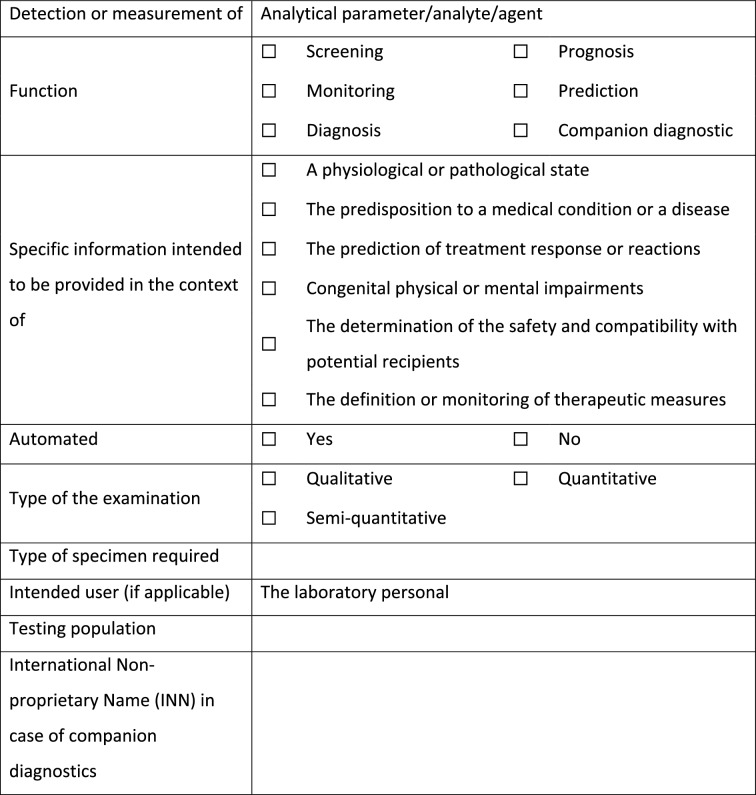
Determination of the Intended Purpose of LDTs Based on the Major Criteria for the Definition of IVD.

The definition of an IVD can be further converted into three questions. By answering these questions as formulated according to Table 3, the task of regulatory demarcation of LDTs from other potential health products and as IVD based on the definition according to the EU IVDR can be performed.

**Table 3:** Regulatory Demarcation of LDTs from Other Potential Health Products and as IVD Based on the Definition According to the EU IVDR.

Question 1	Is the device used in vitro for the examination of specimens derived from the human body?
Question 2	Is the device used for providing one or more of the following information? a. concerning a physiological or pathological process or state b. concerning congenital physical or mental impairments c. concerning the predisposition to a medical condition or a disease d. to determine the safety and compatibility with potential recipients e. to predict treatment response or reactions f. to define or monitoring therapeutic measures
Question 3	Is the device used as specimen receptacle? Note: Specimen receptacle is a device used for the primary containment and preservation of specimens derived from the human body for the purpose of in vitro diagnostic examination
Conditions:	Results
If both the question 1 and 2 are correct	The LDT is an IVD device
If either question 1 or question 2, and question 3 are not correct	The LDT is not an IVD device
If question 3 is correct	The LDT is an IVD device

### Consequences of the LDT Demarcation for Labor Berlin

Based on the developed concept for regulatory demarcation of LDT, the number of CE-marked IVD currently in service at Labor Berlin per department were compared with the number of LDTs currently established within the different departments of the laboratory. The results are presented in Table 4.

**Table 4:** IVD Devices in Service at Labor Berlin per Department (per December 2020).

Laboratory Department of Labor Berlin	IVD devices in service at Labor Berlin	
	Commercially available IVDs	LDTs
Toxicology	39	209
Hematology/Oncology	15	168
Human Genetics	1	145
Laboratory Medicine	274	42
Immunology	20	39
Virology	42	31
Endocrinology	73	24
Microbiology	107	1
Tumor Cytogenetics	10	0
Allergy Diagnostics	3	0
Autoimmune Diagnostics	93	0
Total	677	659

It can be concluded from Table 4 that LDTs constitute nearly 50% of the total IVD devices used in the laboratory. Furthermore, the Toxicology, Hematology/Oncology and Human Genetics departments have the highest number of LDTs in service in comparison with other departments. The low number of CE-marked devices (CE = “conformité europénne”, “European conformity”) used in the field of Hematology/Oncology and Human Genetics also confirms the well-known scarcity of commercially available IVD for rare diseases and common genetic diseases [34].

Of note, the EU IVDR, according to point (d) of Article 5 (5), requires a justification for the use of LDTs that must include the evidence that the target patient group’s specific needs addressed by the LDT cannot be met or cannot be met at the appropriate level of performance by an equivalent IVD device commercially available on the market. In the current absence of any interpretation issued by EU regulatory authorities or the MDCG with regard to the “principle of equivalence” for IVD, this study concludes that the comparison criteria for performance levels should be determined upon the specific patient needs addressed by the testing device. This can include a large variety of analytical and clinical performance characteristics amended by the method principle, the availability of quality controls, turn-around-times and experience from long-term-use of examination procedures, for example.

**Figure 3:**
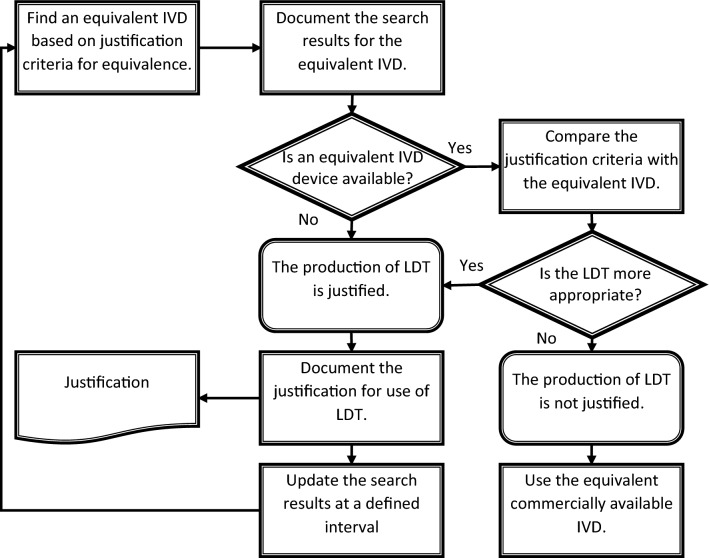
Search for an equivalent CE-marked IVD and justification for use according to Article 5 (5) of the EU IVDR.

It is advisable for the laboratories to create a procedure for the implementation of justification criteria related to LDT use and for the identification of potentially equivalent devices available on the market. According to such a procedure, the laboratory should document the data regarding the availability of potentially equivalent CE-marked devices besides the justification. As a consequence, a laboratory should continuously monitor the market for these IVDs. A scheme for a procedure related to the justification is illustrated according to Fig. 3.

**Figure 4:**
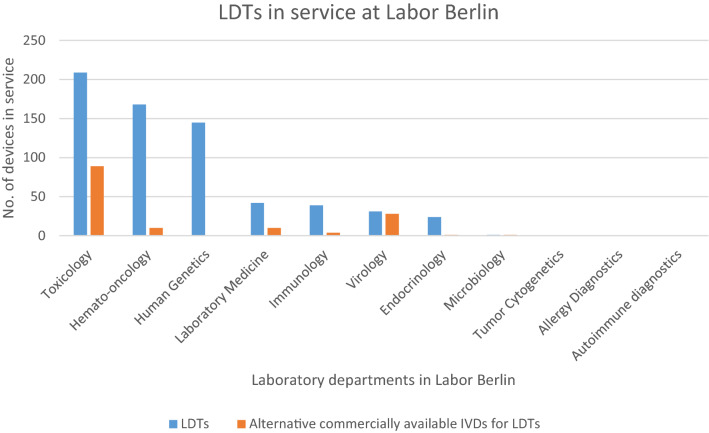
LDTs in service and potential alternatives from commercially available IVD (CE-marked) at Labor Berlin per department.

For Labor Berlin, a preliminary application of the procedure related to the justification for use has identified 143 potentially equivalent CE-marked IVD out of the total of 659 LDTs. This is 21.7% of the LDTs used by Labor Berlin that could possibly be replaced by commercially available devices. As Fig. 4 illustrates, there are departments with examination areas that offer few or no possible alternatives (such as Human Genetics) and departments with a greater choice (such as Toxicology).

### Risk-Based Classification of LDTs

In agreement with international recommendations on risk categorization of IVD, the EU IVDR introduces a risk-based approach for the classification of IVD based on the former GHTF classification system most recently updated by IMDRF [25, 35]. The risk classification shall also be carried out for each LDT that meets the definition of an IVD device according to the EU IVDR. Although this is not explicitly mentioned, the obligation for risk classification can be concluded from the requirement according to Point g) of Article 5 (5) to maintain a specifically detailed documentation for Class D devices. Member States are also allowed to apply this provision to class A, B or C devices.

Therefore, the risk classification shall be performed in accordance with Article 47 and the seven classification rules contained in Annex VIII of the EU IVDR. The classification rules will allow risk classification of the LDT into one of the four risk classes A to D, with class A being the lowest risk and class D the highest. The previously published MDCG document MDCG 2020–2016 offers interpretation with regard to various cases and examples of IVD classification. Apart from commercial manufacturers and notified bodies, MDCG 2020–2016 also explicitly addresses health institutions [36].

The EU IVDR replaces the current list-based classification system of the IVDD with a rule-based system. It is expected that approximately 80% of all products will be assigned to a risk class higher than class A and will therefore be subject to a notified body scrutiny, while under the current IVDD regime this is the case only for about 20% of the devices [37]. It is concluded by this study that this redistribution of IVD risk classes is comparable for LDTs. However, regardless the assigned risk class, the EU IVDR does not require a participation of notified bodies during the conformity assessment of LDTs.

The international perspective on LDT classification confirms a risk-based approach although details of the risk classification concept may differ. In the U. S., the current regulatory framework for LDTs is largely governed by the Clinical Laboratory Improvement Amendment (CLIA] and the central oversight activities by the Centers for Medicare and Medicaid Services (CMS) [38]*.* Five different types of CLIA certificates can be obtained, based on the diagnostics tests performed in the individual clinical laboratory [6]. The risk-based classification into three major risk classes, as it is used for commercial IVD in the U.S., is not applied to LDTs. However, since LDTs are classified as „high complexity tests”, all applicable CLIA requirements must be fulfilled [39]. In view of the “Verifying Accurate Leading-edge IVCT development” (VALID) Act, which was introduced by the U. S. House and Senate lawmakers in 2020, but is currently still not applicable law yet, all types of IVDs including LTDs will be referred to as in vitro clinical tests (IVCTs) [6, 7]. The VALID Act differentiates between “high-risk” and “low risk” IVCTs depending on whether or not an undetected inaccurate result from a test would present a potential unreasonable risk for serious or irreversible harm or death to a patient or would otherwise cause serious harm to the public health [7].

The Australian TGA follows the GHTF recommendations on a risk-based classification scheme for IVD composed of four different risk classes, whereas for LDTs two broad regulatory categories can be distinguished: Classes 1–3 in-house IVD and Class 4 in-house IVDs. Low and middle risk class (Class 1–3) in-house IVD do not need to be included in the Australian Register of Therapeutic Goods (ARTG) but need to comply with certain requirements for the conformity assessment procedure, while manufacturing of Class 4 in-house IVD involves an inclusion into the ARTG and compliance with more stringent requirements related to the conformity assessment procedure [31, 40].

Since Health Canada does not regulate LDTs, there is no national risk classification scheme for LDTs applicable. The classification concept for commercial IVD products follows a risk-based approach consisting of four risk classes from Class I (lowest risk) to IV (highest risk) though [41].

### Basic Elements of Conformity Assessment for LDTs According to the EU IVDR

The third step in the regulatory compliance strategy for LDTs is related to the required elements of conformity assessment.

**Figure 5:**
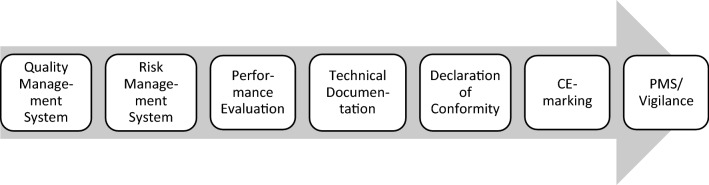
Overview on the general obligations of manufacturers of CE-marked IVD according to Article 10 of the EU IVDR.

For commercially available IVD, Article 10 of the EU IVDR introduces general obligations that a manufacturer shall fulfil regardless of the risk class and of the conformity assessment procedure chosen for the considered IVD product. These general obligations are summarized in Fig. 5.

With the exception of the fulfilment of the general safety and performance requirements set out in Annex I of the IVDR, users of LDTs are generally exempt from all obligations of the regulation. However, a considerable number of conditions according to Article 5 (5) of the IVDR have to be fulfilled by LDT users as prerequisite to apply this “in-house-privilege”. These conditions or requirements and possible implementation methods as suggested by this study are listed and compared to the requirements for CE-marked IVD products in Table 5.

**Table 5:** Overview on the Requirements Related to LDT Use According to Article 5 (5) of the EU IVDR, Proposed Implementation Methods and Comparison with the General Obligations of Commercial IVD Manufacturers According to Article 10 of the EU IVDR.

No	Requirement for LDT use according to Article 5 (5) of the IVDR	Proposed implementation method for LDTs	General requirement for CE-marked IVD as per Article 10, IVDR
I	(a)the devices are not transferred to another legal entity	LDTs shall be manufactured and used only within a single health institution	Not applicable
II	(b) manufacture and use of the devices occur under appropriate quality management systems	QMS according to ISO 15189 and applicable requirements of ISO 13485	QMS according to EN ISO 13485 and QM requirements according to Article 10, IVDR
III	(c) the laboratory of the health institution is compliant with standard EN ISO 15189 or where applicable national provisions, including national provisions regarding accreditation	Compliance with ISO 15189 (as shown by accreditation) or with national provisions, for example the RiLiBÄK (Germany)	Not applicable
IV	d) the health institution justifies in its documentation that the target patient group's specific needs cannot be met, or cannot be met at the appropriate level of performance by an equivalent device available on the market	QM documentation of the justification for use of LDTs. Compare Fig. 3	Not applicable
V	(e) the health institution provides information upon request on the use of such devices to its competent authority, which shall include a justification of their manufacturing, modification and use	QM documentation on the use of LDTs	Technical documentation according to Annex II and III, IVDR
VI	(f) the health institution draws up a declaration which it shall make publicly available, including: (i) the name and address of the manufacturing health institution, (ii) the details necessary to identify the devices, (iii) a declaration that the devices meet the general safety and performance requirements set out in Annex I to this Regulation and, where applicable, information on which requirements are not fully met with a reasoned justification therefore	Documentation and publication of a formal declaration of conformity with Annex I, IVDR	Draw up of an EU declaration of conformity in compliance with Article 17 and Annex IV, IVDR
VII	(g) as regards class D devices in accordance with the rules set out in Annex VIII, the health institution draws up documentation that makes it possible to have an understanding of the manufacturing facility, the manufacturing process, the design and performance data of the devices, including the intended purpose, and that is sufficiently detailed to enable the competent authority to ascertain that the general safety and performance requirements set out in Annex I to this Regulation are met. Member States may apply this provision also to class A, B or C devices in accordance with the rules set out in Annex VIII	Documentation of the design, performance evaluation data and manufacturing process within the laboratory’s QM system	Technical documentation according to Annex II, IVDR
VIII	(h) the health institution takes all necessary measures to ensure that all devices are manufactured in accordance with the documentation referred to in point (g)	Continuous documentation of the data related to manufactured LDTs	Technical documentation according to Annex II, IVDR
IX	(i) the health institution reviews experience gained from clinical use of the devices and takes all necessary corrective actions	Implementation of an LDT review system including CAPA procedures	Technical documentation according to Annex III, IVDR
X	Member States may require that such health institutions submit to the competent authority any further relevant information about such devices which have been manufactured and used on their territory. Member States shall retain the right to restrict the manufacture and use of any specific type of such devices and shall be permitted access to inspect the activities of the health institutions	Control of any documents and records of the QM system related to LDTs	Concepts and methods of market surveillance by competent authorities according to Articles 88ff. IVDR
XI	This paragraph shall not apply to devices that are manufactured on an industrial scale	Laboratories shall not offer their service in the frame of an industrial production chain and environment	Not applicable

It is apparent from Table 5 that a major prerequisite for LDT use is the maintenance of a laboratory’s comprehensive quality management system (QMS) including a documentation providing adequate evidence of the LDT performance characteristics. The QMS goes along with a monitoring and corrective action/preventive action (CAPA) system for LDTs. Of note, LDTs belonging to risk Class D are differentiated from Class A to C LDTs, since a higher level of documentation details must be provided for Class D LDTs in comparison with lower risk classes.

While—not surprisingly—many elements related to organizational conditions for LDTs are not applicable to CE-marked IVD products, the QMS and documentation requirements for LDTs show some degree of similarity with the general obligations related to the QMS and the technical documentation of CE-marked products. However, applicable standards (ISO 15189 [42] versus ISO 13485 [43]) are not interchangeable and the extent of the required documentation is supposed to be less detailed for LDTs than for CE-marked devices.

Although ISO 15189 is specifically mentioned by the IVDR as reference for an appropriate QMS of laboratories using LDTs, the comparison according to Table 6 provides an overview on the degree of conformity of EN ISO 15189 compliant laboratories with the regulatory obligations for LDTs according to Article 5 (5) of the IVDR. It may be seen that ISO 15189 compliance only partially covers the requirements of the IVDR for the manufacture of LDTs.

**Table 6:** Cross-References Between Regulatory Obligations for Manufacture of LDTs According to the EU IVDR and Requirements According to ISO 15189.

Applicable regulatory obligations according to the IVDR	EU IVDR references to the requirements		Compliance status	Reference to ISO 15189
	Related article	Related annex		
General safety and performance requirements	Article 5 (5)	Annex I	Partial	Sections 5.4, 5.5, 5.6
Quality management system	Article 5 (5) (b) & (c)		*Complete*	Section 4.2
Risk management system		Annex I (2, 3, 4 & 5)	Partial	Section 4.14.6
Performance evaluation		Annex I (9)	Partial	Section 5.5
Justification	Article 5 (5) (d)		No	
Documentation for compliance	Article 5 (5) (g)		*Complete*	Sections 4.2.2 and 4.13
Publicly available declaration	Article 5 (5) (f)		No	
Surveillance system (review of experience)	Article 5 (5) (i)		*Complete*	Sections 4.8, 4.9 and 4.14, 5.3
Surveillance system (corrective actions)	Article 5 (5) (i)		*Complete*	Sections 4.10 and 4.11, 5.3
Transfer of LDTs	Article 5 (5) (a)		No	
Scale of production	Article 5 (5)		No	

### Conduct of the Regulatory Approval Procedure for LDTs

According to the EU IVDR, LDTs are exempt from all regulatory obligations, but have to fulfil the applicable general safety and performance requirements (GSPR) set out in Annex I of the IVDR. In conclusion, LDTs, regardless of their risk class, do not require CE-marking, are exempt from any involvement of notified bodies within the conformity assessment and do not have to follow any of the conformity assessment procedures described for commercially available IVD products according to Article 48 of the IVDR. Since conformity of the LDT with the GSPR nevertheless must be assessed, this procedure conducted by a laboratory could be defined as a “simplified” conformity assessment procedure.

According to the IVDR, the GSPR are grouped into three different chapters:

1. Chapter I: General requirements;
2. Chapter II: Requirements regarding performance, design and manufacture;
3. Chapter III: Requirements regarding the information supplied with the device.

In our study, Labor Berlin developed a GSPR checklist to demonstrate the evidence of compliance with the GSPR relevant for each LDT (or group of LDTs, if grouping was possible). The structure of the GSPR checklist is shown in Table 7 and is an adaptation of the original GHTF “Essential principles” checklist used for commercial IVD products [27].

**Table 7:** Structure of the GSPR Checklist to be Filled for Evidence of Compliance with Annex I of the EU IVDR.

No	General safety and performance requirement according to Annex I, IVDR	A/NA	Method used to demonstrate conformity	Method reference	Reference to the identity of controlled documents within the QMS	Justification for applicability/non-applicability
1	Devices shall achieve the performance intended by their manufacturer and shall be designed and manufactured in such a way that, during normal conditions of use, they are suitable for their intended purpose. They shall be safe and effective and shall not compromise the clinical condition or the safety of patients, or the safety and health of users or, where applicable, other persons, provided that any risks which may be associated with their use constitute acceptable risks when weighed against the benefits to the patient and are compatible with a high level of protection of health and safety, taking into account the generally acknowledged state of the art	A	Compliance with international standards	ISO 15189 (whole standard); ISO 22367, 5.4, 5.5, Annex A.4, Annex D; ISO 13485, 7.3	Quality manual, documented procedures for the design and development process for LDTs including validation and verification procedures	Requirement is fully applicable for LDTs, because performance and safety are essential throughout the lifecycle of each LDT
…	…	…	…	…	…	…

Only few IVDR requirements can be fully covered (italic) by compliance with ISO 15189

The checklist is applied to the General Requirement No. 1 of Annex I of the EU IVDR. A method used to demonstrate conformity could be, for example, the application of an international standard, specifications etc. The method reference is, for example, the title of a standard. The reference to the QMS could be the identifier of an SOP, for example

*A* applicable requirement, *NA* not applicable

Even though the general requirements according to Chapter I of Annex I are applicable to every LDT, the applicability of the specific requirements given in Chapter II and III largely depends on the type of the considered LDT.

### Risk Management for LDTs

Annex I of the IVDR requires the implementation and maintenance of a documented risk management system with a detailed risk management process at hand. Although Sect. 4.14.6 of the standard EN ISO 15189 demands medical laboratories to evaluate the impact of potential failures on examination results and to modify examination processes accordingly, this standard alone does not assume to cover the detailed requirements of a risk management system according to Annex I of the IVDR. In contrast, the previously published standard ISO 22367 provides medical laboratories with a framework for handling risks related to medical laboratory examinations [44]. ISO 22367 guides medical laboratories to identify, estimate, evaluate, control, and monitor the risks to patients and laboratory personnel related to their medical laboratory examinations, including the pre- and post-examination phases. In addition, the application scope of ISO 22367 also covers those medical laboratories that manufacture and use LDTs. Therefore, health institutions can use the standard ISO 22367 to comply with the requirements of a risk management system as required by the IVDR. However, ISO 22367 advocates a risk management process for handling risks related to all examinations of a medical laboratory. According to the IVDR, a risk management system is only mandatory for the medical laboratory examinations that involve the use of LDTs. To ensure efficient usage of available resources and to reduce the load of documentation on health institutions, it is therefore advisable to adapt ISO 22367 for a risk management system with the application scope limited to those medical laboratory examinations involving LDTs.

### Performance Evaluation of LDTs

Performance evaluation is a central element of any conformity assessment procedure for IVD since the fulfilment of performance requirements is demonstrated through the appraisal and assessment of relevant performance data of the IVD device in focus [45].

Annex I of the IVDR explicitly refers to both the analytical performance and the clinical performance of a device and requires that, while taking account of the generally acknowledged state of the art, the device shall achieve the performances as stated by the manufacturer. With the newly introduced legal definition of the term “performance evaluation”, the EU IVDR also emphasizes the relevance of the so-called “scientific validity” of an analyte that should be verified by performance evaluation to provide for sufficient clinical evidence pertaining to a device.

Neither the IVDR nor official EU guidance such as, for example, MDCG guidance currently specify the necessary documentation for performance evaluation of LDTs. Standards such as ISO 20916 [46] and the more ancient EN 13612 [47] include requirements for the conduct of clinical performance studies and for performance evaluation in general, but do not address the specific circumstances of LDT use. However, the requirements given under Article 56 and Annex XIII of the EU IVDR can work as a certain basis for the performance evaluation of LDTs and may be adapted in a simplified way for the needs of medical laboratories using LDTs. For Labor Berlin, the entire task of performance evaluation and its subsequent documentation was structured into four phases as shown in Table 8.

**Table 8:** Phases of Performance Evaluation of LDTs as Adapted from Annex XIII of the EU IVDR.

Phase of the performance evaluation	Title of the phase	Performance evaluation activities
Phase I	Planning of performance evaluation	Determination of the scope of the performance evaluation; description of the device under evaluation; assignment of responsibilities for the performance evaluation; selection of relevant performance criteria to generate the necessary clinical evidence for the LDT
Phase II	Execution of performance evaluation	Identification of the scientific validity and relevant analytical and clinical performance data
		Generation of data through performance studies, if applicable
		Appraisal of relevant performance data
		Assessment of relevant performance data
Phase III	Documentation of performance evaluation	Documentation of the results of performance evaluation
Phase IV	Update of performance evaluation	Post-market performance follow-up, if applicable

The requirements for performance evaluation of LDTs were adapted in such a way that the extent of the documentation changes according to the novelty of the technology involved and the risk class of the LDT device. This is in agreement with segregation of IVD for performance evaluation into the following three different categories according to GHTF: Established and standardized tests, established and non-standardized tests, novel tests [45].

### Key Characteristics of International LDT Regulation

The regulatory strategy developed in this study was considered in the view of international approaches for LDT regulation in a number of well-established regulatory markets. A high-level comparison on the current approaches in the EU, U. S., Australia and according to the Canadian standard Z316.8-18 identified a number of key characteristics that are common in all or many markets with regard to the setting of safety and performance requirements for LDTs. These elements are listed according to Table 9. As a result, major characteristics of LDT regulation include provisions for a comprehensive QMS and risk management system, requirements for performance evaluation/validation and related documentation and a certain monitoring across the life cycle of LDTs. There is, however, neither a common understanding on the standards to be applied nor on the extent of regulatory oversight including registration or notification procedures.

**Table 9:** Key Characteristics as Central Elements for LDT Regulation in Different Advanced Regulatory Markets.

No	Element	EU IVDR	U.S	Australia	Canada
1	Quality management system	*Yes* *ISO 15189 or further national provisions*	*Yes* *CLIA, 21 CFR 820*	*Yes* *ISO 15189; ISO 17025* *ISO 13485 (for class 4)*	Yes (Section 4)
2	Risk-based approach for classification/categorization	*Yes* *(4 classes A–D)*	*Yes* *(according to “complexity “and” high/low level risk”)*	*Yes* *(4 classes 1–4)*	No
3	Risk management system	*Yes*	*Yes*	*Yes*	Yes (Section 5.4)
4	Evaluation and documentation related to essential requirements for quality, safety, performance	*Yes*	*Yes*	*Yes*	Yes (Sections 5, 6, 7)
5	Product monitoring and surveillance	*Yes*	*Yes*	*Yes*	Yes (Section 7.4)
6	Register	No	No	*Yes*	Not appplicable
7	Justification for use	*Yes*	No	No	Not appplicable
8	Notification requirement	No	*Yes*	*Yes*	Not appplicable
9	Regulatory oversight mechanism	*Yes*	*Yes*	*Yes*	Not appplicable

For Canada, only the sections of the standard Z316.8-18 are considered. Elements that involve interactions with a regulatory body (e. g. registration, notification etc.) are therefore deemed to be not applicable for Canada. The boxes italic indicate common elements

## Discussion

In view of the new European regulatory provisions for IVD including harmonised requirements for IVD manufactured and used within European health institutions, this study suggests a regulatory strategy for compliance of LDTs within the frame of a large, academic European medical laboratory. As Labor Berlin is accredited according to ISO 15189 and therefore has a full quality management system (QMS) in place, the strategy presented in this study does not only include a regulatory concept, but also demonstrates how regulatory requirements for LDTs can be integrated into the quality framework of an accredited laboratory.

The strategy based on four major steps reveals a number of critical issues to be resolved by both the regulatory framework provided for LDTs and by the health institutions that have to implement the regulations.

A major prerequisite for regulatory compliance in the context of LDTs is a sound and unambiguous definition of the scope of the regulatory framework. There should be no uncertainty about what is understood as LDT and what is therefore covered by the regulatory framework and what—in contrast—is outside the scope of the framework. In the current absence of any clear definition by the EU legal framework or any interpretation by EU official committees such as the Medical Device Coordination Group (MDCG), this study suggests four major scenarios for the qualification and demarcation of LDTs that are solely based on the intended purposes of use as defined by the laboratory as LDT user. This is in compliance with further non-EU legal and standards requirements that are considered in this study. It is also in agreement with the central quality and safety concept globally acknowledged for medical devices centered around the intended purpose of use of a device that determines subsequent steps of the regulatory approval procedure. However, a controversy debate is currently performed under medical laboratory professionals in the EU that reflects the current uncertainty in the field of LDT definition and demarcation. In this regard, it is even suggested that products for general laboratory use may not be considered in-house IVD if used in a complex medical examination procedure [16].

For Labor Berlin, the evaluation of laboratory methods revealed that LDTs constitute nearly 50% of the total IVD devices used in the laboratory. This high ratio confirms the significance of LDTs for patient care, especially in cases where individualized testing becomes more and more relevant and where commercial alternatives are not available or will disappear from the market due to the new legislation. The ratio of LDTs estimated in this study is in agreement with most current study results of other academic laboratories in the EU [48].

As pointed out in this study, the justification for use of LDTs is a unique requirement set by the EU IVDR. The major problem here is the missing definition of the limits and criteria to be used for the justification in the context of device equivalence. Medtech Europe and the British MHRA have developed some opinion on this aspect, but an official EU interpretation is currently not available [18, 49]. An additional problem is the general lack of data available for laboratories about the performance characteristics of commercially available IVD. Without these performance data, it is nearly impossible to demonstrate equivalence between devices. This problem will only be partially solved by the new Summary of Safety and Performance according to Article 29 of the EU IVDR, since this summary will be only required for Class C and D products. Given the highly individual technological and diagnostic specifications of LDTs, it may be concluded that comparison criteria for performance levels should be determined upon the specific patient needs addressed by the testing system. Finally, these can only be considered by medical laboratory professionals in view of the individual clinical situation of the patient. Guidance on potential criteria for IVD equivalence and the justification for use would however increase transparency in this regard.

The requirement for the QMS constitutes a major element in the context of LDT regulation by the EU IVDR. However, the elements of the QMS are not clearly defined in the IVDR. Although the standard EN ISO 15189—which is internationally regarded as the “gold standard” for quality and competence in medical laboratories—is mentioned and even constitutes a harmonized European standard in the context of the Regulation (EC) 765/2008 [50], applicable national provisions seem to be at least equivalent with ISO 15189. In Germany, for example, the so-called RiLiBÄK sets requirements for the QMS and for the quality assurance for medical laboratory examinations, which are similar, but far from being identical to ISO 15189 in many aspects [51]. It is therefore debated in which way presumption of conformity to the requirement with regard to the QMS will be concluded for the RiLiBÄK or any other national provisions in this field [17].

Furthermore, compliance with ISO 15189 primarily refers to the requirement for using the LDTs under an appropriate QMS, but does not focus on the requirements for manufacturing of the LDTs. It can, however, be argued that additional elements regarding the design and manufacturing of the devices are required in the context of such a QMS. Section A.4 of the risk management standard ISO 22367, therefore, advocates the use of the approach described in Section 7.3 of the standard ISO 13485 for design and development activities related to LDTs [43, 44].

Similar to the QMS, the requirements related to the risk management system in the context of LDTs are not yet sufficiently specified within the EU. With the new Annex I requirements, the EU IVDR introduces an ambitious approach demanding an iterative process including planning, analysis, evaluation, control and continuous review related to all kinds of product risks. In this regard, the EU requirements even exceed the sophisticated Australian framework for LDTs.

Both the Australian and the EU legal framework explicitly demand the health institutions to demonstrate compliance with the GSPR and prepare a documentation including the design and performance data of LDTs. However, the EU IVDR does not provide information on the extent of the documentation required for LDTs compared to the extent given in Annex II of the IVDR for commercial IVD. Questions arise especially with regard to performance evaluation where the EU IVDR does not mention whether to produce documents related to the planning, appraisal and assessment of the data, including the post-market performance evaluation and the retention period for maintaining records of the documentation. It is questionable whether compliance with ISO 15189 alone is sufficient for the fulfillment of most of the GSPR as recently suggested [17]. In this study, a practical adaptation from the IVDR requirements according to Annex XIII is proposed for performance evaluation of LDTs that takes into account the resources of the specific laboratory environment in which LDTs are manufactured.

With regard to transparency and traceability, especially Australia requires the notification and registration of all LDTs in specific databases, whether official or in-house, whereas only the information of the Class 4 in-house devices is publicly available. This is considered to be a very crucial step which is currently not established in the EU at all.

As the comparison of the regulatory framework for LDTs among major markets revealed, LDTs are mostly considered by either national primary and secondary legislation or by national standards. However, there is currently no harmonized approach for LDT regulation at the international level available. Although the IMDRF most recently addressed specific topics related to personalized medical devices, personalized in vitro diagnostic testing in the context of LDTs is not within the scope of these recommendations [52]. The comparison between the existing legislation and standards provided in this study suggests a set of common and essential criteria for LDTs as most significant regulatory elements for ensuring quality, safety, and performance of LDTs. These elements may be considered as characteristics of a kind of “best-practice approach” of potential international recommendations on LDT regulation.

Especially under consideration of the effect of LDTs on innovation in medical laboratory testing and their relevance in the management of global emergencies, a balanced view on LDT regulation is necessary. On the one hand, requirements as set by the EU IVDR for LDTs intend to provide a high level of health protection for patients in parallel to allowing the laboratories to manufacture LDTs that address specific needs of patient groups or even individual patients. Of note and in contrast to Australia, the EU does not even require any third-party participation in the conformity assessment procedure of high-risk class LDTs (Class D or Class 4 according to the EU and Australian system, respectively),—an aspect bearing chances and risks at the same time. On the other hand, compliance with the GSPR and additional obligations put a significant burden on medical laboratories. Especially with the new EU requirement regarding the justification for use of LDTs, the health institutions might hesitate to invest their resources in the development or maintenance of novel tests with unmet medical need. These circumstances may represent a serious obstacle to innovation and thus limit patient access to potentially beneficial or even essential diagnostics.

With regard to the competent authorities of the different EU Member States, new surveillance tasks will have to be fulfilled in order to determine whether the health institutions and the LDTs manufactured by them are compliant with the requirements of the EU IVDR. A guidance from the MDCG Subgroup “Market Surveillance” expected to be published with regard to “In-house manufacturers” during the year 2021 should bring more certainty and transparency for a harmonized interpretation of the IVD obligations [53].

## Conclusions

By including IVD manufactured and used only within health institutions in the regulatory framework of the new EU IVDR, medical laboratories are confronted with new and EU-wide requirements for LDTs. On the one hand, these requirements are reduced to a significant extent in comparison to commercially available IVD because LDTs are exempt from CE-marking according to formal conformity assessment procedures. On the other hand, certain conditions have to be fulfilled by LDT manufacturers that primarily focus on a comprehensive QMS integrating numerous further elements related to risk management, performance evaluation and device monitoring including its documentation.

The application of a stepwise approach based on four internationally recognized parts of conformity assessment as introduced in this study has defined a regulatory strategy for compliance with LDT requirements given by the EU IVDR. However, many unanswered questions and challenges in the implementation process remain to be resolved, especially with regard to the scope of LDT definition, the correct application of standards and the required extent of documentation for LDTs. In order to strengthen the European harmonization process, this should preferably be done on the level of MDCG guidance. During guidance development, it will be beneficial to consult with European medical-scientific societies and laboratory professionals to consider adequately the needs of individual patient care and testing. European and international standards, as applicable, should also be considered in order to provide transparent and reliable guidance for the interpretation of regulatory details with regard to LDTs.

On a global level, however, there is currently no international consensus for essential requirements for quality, safety and performance of LDTs available. Adequate platforms for such consensus documents or recommendations could be delivered by IMDRF or by the International Organization for Standardization (ISO). An international standard addressing specific requirements for the design, development, and validation of LDTs would be helpful. The National Standard of Canada Z316.8-18 certainly defines a milestone in this context but would need to be amended in key aspects, for example with regard to performance evaluation and life cycle management of LDTs. The key characteristics identified in this study as common criteria for ensuring quality, safety and performance of LDTs may be considered as central elements of international consensus guidance.
